# Prevalence of bluetongue virus infection and associated risk factors among cattle in North Kordufan State, Western Sudan

**DOI:** 10.1186/1746-6148-10-94

**Published:** 2014-04-24

**Authors:** Ibrahim A Adam, Mohamed A Abdalla, Mohamed EH Mohamed, Imadeldin E Aradaib

**Affiliations:** 1 Molecular Biology Laboratory, Department of Clinical Medicine, Faculty of Veterinary Medicine, University of Khartoum, P.O. Box 32 Khartoum North, Sudan; 2 Department of Preventive Veterinary Medicine, College of Veterinary Medicine, Sudan University for Science and Technology, Khartoum, Sudan; 3 Veterinary Medicine Department, College of Food and Agriculture, United Arab Emirates University, Al Ain, United Arab Emirates

**Keywords:** Epidemiology, Survey, Orbiviruses, BTV, ELISA, Sudan

## Abstract

**Background:**

Bluetongue virus causes febrile disease in sheep and a fatal hemorrhagic infection in North American White-tailed deer. However, in cattle the disease is typically asymptomatic and no clinical overt disease is associated with bluetongue infection. Bluetongue virus activity has been detected in Khartoum, Sennar and South Darfur states of the Sudan. Currently, no information is available in regard to previous exposure of livestock to Bluetongue virus in North Kordufan State, the largest livestock producing region in the country. The present study was conducted to determine the prevalence of bluetongue antibodies and to identify the potential risk factors associated with the presence of bluetongue antibodies among cattle in North Kordufan State, Sudan. A total of 299 bovine blood samples were collected randomly from six localities in North Kordufan State and were tested by enzyme-linked immunosorbent assay (ELISA) for detection of BTV-specific immunoglobulin G (IgG) antibodies.

**Results:**

The serological evidence of Bluetongue virus infection was observed in 58 out of 299 cows, accounting for a 19.4% prevalence rate among cattle in North Kordufan State. Older cattle (>2 years of age) had four times the odds to be infected with BTV compared to young cattle (OR = 4.309, CI = 1.941-9.567, p-value = 0.01). Application of preventive measures, such as spraying or dipping with insecticide protects cattle against Bluetongue infection. Application of vector control measures decreased the odds for bluetongue seropositivity by 7 times (OR = 7.408, CI = 3.111-17.637, p-value = 0.01).

**Conclusions:**

The results of this study indicated that age and application of routine insecticides are influential risk factors for seroprevalence of Bluetongue in cattle. Surveillance of Bluetongue virus should be extended to include other susceptible animals and to study the distribution of the insect vectors in the region to better predict and respond to BTV outbreak in the State of North Kordufan, Sudan.

## Background

Bluetongue virus (BTV) is a double stranded (ds) RNA orbivirus of the family Reoviridae [1-3]. To date, there are 26 distinct BTV serotypes distributed worldwide. BTV serotypes 25 and 26 were isolated and identified recently [4,5]. In Sudan, previous studies have shown that the seasonal incidence of BTV is a predictable event related to the rainy season [6]. BTV is transmitted by different species of *Culicoides* midges, with *Culicoides imicola* being the principal vector of BTV in the Sudan [6-9]. At least, five BTV serotypes including serotypes 1, 2, 4, 5 and 16 are enzootic in different regions of the Sudan as these serotypes were recovered from sentinel calf herds at Khartoum University farm, Shambat; and from Nyala, South Darfur State, as well as from Umbenin, Sennar State, Sudan [6-8,10,11].

BTV causes febrile disease in Sudanese breeds of sheep and a fatal hemorrhagic disease in North American white-tailed deer [12,13]. In cattle, goats and camels, the infections are usually inapparent and evidence of clinical disease is seldomly observed. However, indirect losses associated with loss of body weight and condition, drop in milk production, and poor subsequent reproductive performance were thought to have greater economic effect than occasional overt disease [10,14-16]. In addition, there is restriction on the international trade of livestock and associated germplasm from BTV-endemic countries, unless the animals are certified free of infection by conventional virus isolation or serology [14,17-19]. The restriction leads to economic losses for BTV-endemic countries, like Sudan, which rely on the sale of livestock for foreign exchange.

Currently, there is no information available about the prevalence and the potential risk factors associated with the disease in different States of Sudan. Previous studies on experimental or sentinel cattle herds for BTV infection showed that infected cattle developed viremia and became seroconverted. Thus, cattle play an important role in the epidemiology of BTV as virus reservoir for transmission by insect vectors to highly susceptible sheep [6,20,21]. Earlier serologic studies for the presence of BTV-specific antibodies and subsequent virus isolation attempts have been described in cattle in various regions of Sudan [7,8,10,22,23]. Therefore, it is becoming increasingly obvious that the control of orbiviruses, particularly BTV, is especially important in the Sudan given the large numbers of livestock in the country, and their importance to the national economy and rural communities. The epidemiologic studies including implementation of improved surveillance are urgently needed to better predict and respond to this devastating disease in the Sudan [24]. Most of the existing data about the epidemiology of BTV in the Sudan are relatively old.

The objectives of the present study were to estimate the prevalence of BTV infection and to identify the potential risk factors associated with BTV infection among cattle in North Kordufan State, Sudan.

## Methods

### Study area

**Figure 1:**
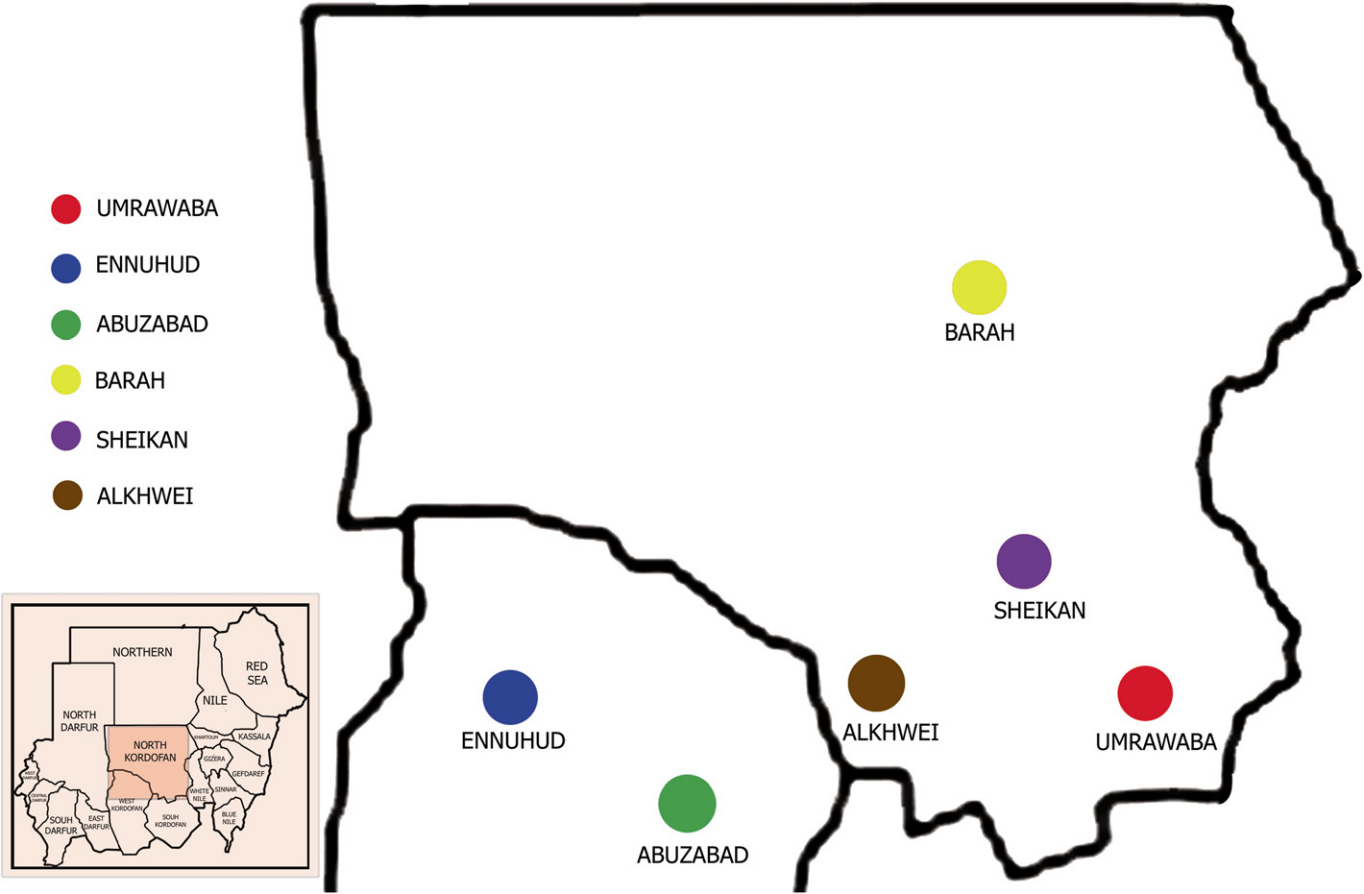
Map of different localities in North Kordufan State, Sudan.

The North Kordofan State is located between longitudes 27.00 and 32.20 East and latitudes 12.12° and 16.4° North, occupying an area of 190,840 square km. The State is boarded by North Darfur State in the north-west; Northern State in the north; Khartoum State in the east; White Nile State in the south-east; West Kordofan state in the south-west and South Kordofan State in the south. The total human population in North Kordufan is approximately 2.9 million. About 63%, 24% and 13% are rural, urban and nomadic people, respectively. The livestock population in North Kordufan constitutes one of the major sources of the income to rural communities and the national economy. Recent official livestock population was estimated to be 960, 500 for cattle; 7,200,000 for sheep; 3,600,000 for goats; and 1,200,000 for camels. The map of North Kordufan showing the six localities included in the study is presented in (Figure 1).

### Study design

A cross sectional study was conducted to estimate the prevalence rate of BTV in cattle, North Kordufan, and to investigate the potential risk factors associated with the disease. The multistage random sampling method was conducted. Six localities in North Kordufan State were randomly selected from all nine localities in North Kordufan (Figure 1). Two administration units were selected randomly from each locality. Seven villages were selected from each unit. Finally, simple random sampling was applied to choose the cattle from each herd [25].

### Questionnaire

A pre-tested structured questionnaire with the primary objective of elucidating the multifactorial background of disease was conducted in an interactive manner at all selected herds. All animals included in this study were subjected to a questionnaire, which was filled out by the animal owners. The questionnaire included individual risk factors attributes age (younger animals < 2years, older animals 2 years and above), sex (male, female), breed (indigenous, exotic), body condition (emaciated, fats). The management risk factors attributes include herd size (small, medium and large), grazing system (nomadic, seminomadic and stationary), milk production (high, low), vector control (use of insecticide or not), the source of each animal in the herd (raised on farm, purchased from other farms or purchased from local market), presence of other animal species in the herd, (the presence of other animals such as sheep, goat and camels in the cattle herd). Herd size of cattle (small, medium and large) and farm yard (cattle kept indoors or outdoors). The original version of the questionnaire is provided as an Additional file 1.

### Ethical clearance

The blood collection procedure from cattle was performed by qualified veterinarians following proper physical restraint of animals to ensured both personnel and animal safety. Livestock owners were explained the study purposes and procedures and upon agreeing to participate they provided a written consent prior to study procedures and blood collection from their animals. The study received ethical clearance from the Research Board of the College of Veterinary Medicine, Sudan University for Science and Technology, Khartoum, Sudan. The risk factor information was obtained from the animal owners through the questionnaire form, which permitted use of the samples for diagnostic and research purposes.

### Collection of blood samples

A total of 299 serum samples of cattle were collected randomly from six localities in North Kordofan State, Sudan. These localities include (Umrawaba, Barah, Sheikan, Ennuhud, Elkhuwei and Abuzabad). Blood samples were collected from the jugular vein in clean sterile vaccutainers and were allowed to clot and sera were separated and kept frozen at -20°C until used for detection of BTV-specific Ig G antibodies.

### Enzyme-linked immunosorbent assay (ELISA)

Indirect enzyme-linked immunosorbent assay (ELISA) was performed to screen the sera for BTV-specific immunoglobulin G (IgG) antibodies basically as described by [6]. ELISA was performed in 96-well immunoassay microplates (Nunc, Roskilde, Denmark) and optimal working dilutions of reagents were determined by chessboard titration. Unless stated otherwise, 100 microlitres (ul) test volumes were used in the ELISA assay. The incubations were performed for 1 h at 37°C. The plates were washed three times with PBS containing 0 I% Tween 20 (Merck, Darmstadt, Germany) (PBST), wells were post-coated with 200, ul of PBS containing 2% bovine serum albumin (Calbiochem, La Jolla, USA), and the diluent for reagents was PBS containing 10% skimmed milk (Amba, Denmark). Briefly, the plates were coated with BTV antigen and incubated overnight at 4°C. The source of the antigen used is BHK-infected BTV prototype serotype 1 (BTV-1). The details for the preparation of the BTV antigen were described previously by Mohamed [26]. The plates were washed, and aliquots of test sera (positive and negative controls) were added in separate wells at a dilution of 1:100. After a 1-h incubation, the plates were washed, and rabbit anti-bovine IgG conjugated with horse radish peroxidase (HRP) was added to the plate at a dilution of 1: 2,000 and incubated for 1 h. The plates were then washed and the substrate, 2,2′-azino-bis(3-ethylbenthiazoline-6-sulfonic acid, (Kirkegaard and Perry Laboratories) was added. The results were read by using ELISA reader set at 405 nm. A presumptive diagnosis was made when Ig G antibody in the test sample had a significant colour change or had higher optical density than the ratio between the positive and negative controls.

### Statistical analyses

The data were entered in computer using statistical package for social studies (SPSS) software package for window (version 16.0) and double checked before analyses. Univariate analysis using Chi-square test was conducted for the association between the potential risk factors and BTV seropositivity. The results of the univariable analysis were reentered in the final model using ultivariable analysis. A multivariable model for the outcome variable was constructed using manual stepwise forward logistic regression analysis. BTV infection was considered as the dependent variable and the risk factors as independent variables. Finally, odd ratios and 95% confidence interval (CI) were calculated, and risk factors with a p-value < 0.05 were taken as significant association.

## Results

The serological evidence of BTV infection was observed in 58 out of 299 cows accounting for a 19.4% prevalence rate among cattle in North Kordufan State. The highest and the lowest rate of infections were recorded in Shiekan and Elnnuhud, (15%) and (27.5%), respectively. Initially, univariate analysis using Chi-square test was conducted for the association between the potential risk factors and BTV infection. The results of the univariate analysis are presented in (Table 1). The results obtained from the univariable model were re-entered into a final multivariate model using logistic regression analysis. In the final models, a variable with a P-value <0.05 was considered statistically significant. The individual risk factors attributes indicated that older cattle (>2 years of age) were four times more likely to be infected with BTV (OR = 4.30, CI = 1.941-9.467, p-value = 0.01). Weak and emaciated cattle were almost 3 times more likely to be at risk for contracting BTV infection (OR = 2.925, CI = 1.146-7.606, p-value = 0.025). The management risk factor attributes showed that the preventive measures, such as routine application of insecticide in the selected herds and spraying or dipping decreased the odds for bluetongue seropositivity by 7 times compared to non sprayed cattle (OR = 7.408, CI = 3-111-17.637, p-value = 0.01). In contrast, there was no significant difference between BTV seropositive cattle and other individual or management risk factors included in the study such as, animal sex, animal source, grazing system, other animals in the herd, herd size, farm yard, milk production, history of diseases and localities. The results are shown in (Table 2).

**Table 1:** Univariate analysis for the association between BTV infection and the potential risk factors

**Risk factors**	**Animals tested**	**Animals affected (%)**	**df**	***χ*** **2**	**p-value**
Locality			5	4.181	0.524
Abuzabad	57	14(24.6%)			
Um Ruwaba	58	9(15.5%)			
Bara	36	7(19.4%)			
Shiekan	60	9(15%)			
Ennuhud	40	11(27.5%)			
Elkhuwaye	48	8(16.7%)			
Age			1	13.03	0.001
Young	119	11(9.2%)			
Old	180	47(26.1%)			
Sex			1	1.99	0.159
female	262	54(20.6%)			
male	54	4(10.8%)			
Breed			1	0.399	0.368
Indigenous	272	54(19.9%)			
Cross	27	4(14.8%)			
Body condition			1	5.491	0.012
Bad	234	52(22.2%)			
Good	65	6(9.2%)			
Animal source			2	0.165	0.921
-Raised on farm	213	41(19.2%)			
Purchased from other farm	8	2(2.5%)			
purchased from local market	78	15(19.2%)			
Grazing system			2	1.097	0.578
Stationary	73	15(20.5%)			
Nomadic	50	12(24%)			
Semi nomadic	176	31(17.6%)			
Herd size			2	1.321	0.517
Small	66	16(24.2%)			
Medium	98	17(17.3%)			
Large	135	25(18.5%)			
Vector control			1	18.506	0.001
No	167	47(28.1%)			
Yes	132	11(8.3%)			
Other animal			1	0.189	0.387
No	152	28(18.4%)			
Yes	147	30(20.4%)			
Farm yard			1	0.259	0.358
Outside	115	24(20.9%)			
Indoor	184	34(18.5%)			
Milk production			2	0.680	0.712
No	159	33(20.8%)			
Low	131	24(18.3%)			
High	9	1(11.1%)			

**Table 2:** Multivariate model using logistic regression analysis for the association between the potential risk factors and BTV seropositive cattle in North Kordufan State, Sudan

**Risk factors**	**OR**	**95% C.I**	**P-Value**
**Age**	Ref		
Young	4.30	1.94-9.57	0.01^*^
Old			
**Vector control**	Ref		
Yes	7.41	3.11-17.64	0.01^*^
No			
**Body condition**	Ref		
Emaciated	2.95	1.15-7.60	0.03^*^
Fat			
**Localities**	Ref		
Abuzabad	0.06	0.06-1.28	0.74
Umrawaba	2.09	0.29-40.64	0.46
Bara	0.08	0.01-2.47	0.15
Sheikan	0.24	0.01-4.44	0.33
Ennuhud	0.77	0.08-7.45	0.82
Elkhuwaye			
**Sex**	Ref		
Female	0.38	0.11-1.24	0.11
Male			
**Breed**	Ref		
Indigenous	0.96	0.06-50.95	0.98
Cross			
**Animal source**	Ref		
Purchase from local market	1.37	0.44-4.23	0.59
Raised on farm	0.86	0.08-9.27	0.90
Purchase from other farm			
**Grazing system**	Ref		
Stationary	1.91	0.20-17.83	0.57
Nomadic	0.69	0.07-6.78	0.75
Semi nomadic			
**Herd size**	Ref		
Small	2.79	0.61-12.86	0.19
Large			
**Farm yard**	Ref		
Indoor	2.79	0.62-14.28	0.17
Outdoor			
**Milk production**	Ref		
No	0.63	0.32-1.24	0.18
Low	0.15	0.01-1.89	0.14
High			
**Other animals**	Ref		
No	1.20	0.45-3.20	0.73
Yes			

p-value* <0.05 was considered significant.

## Discussion

Bluetongue disease constitutes one of the major veterinary problems in sheep and North American white-tailed deer [12,13]. However, in focal areas of endemicity, goats and cattle develop subclinical infection [6,8,10]. In our laboratory, a lot of research efforts have been made to facilitate rapid molecular detection and differentiation of orbiviruses, including BTV, in susceptible native breeds of livestock [11,13,15,17,24,27-33].

Previous epidemiological surveys showed high prevalence rates for BTV infections in Iran (93.5%) and Southern Turkey (88%) [34,35]. In Sudan, earlier serological surveys indicated that BT infection is generally widespread and occurs in all domestic ruminants, with as high as 61.5% prevalence rate among sheep in Juba, South Sudan [22]. A subsequent serological survey for antibodies against bluetongue in cattle in Khartoum State showed high prevalence rate of 51.1% among the examined animals [23]. In the present study, a seroprevalence of BTV infection in cattle of North Kordufan (19.4%) is markedly lower than previously reported prevalence rates compared to other states of Sudan. In this study, the prevalence of BT disease is comparable to those reported amongst sheep in El-Dien (20%), Western Sudan, and Atbara (21.9), Northern Sudan [22]. The presence of BT disease in Sudan and the risks these infected cattle pose for native breeds of sheep, necessitate the importance of an improved surveillance system for this viral pathogen in Sudan. In the present investigation, the final models of BTV seropositive cattle indicated that only three independent risk factors were statistically significant. When assessing age as risk factor, there was a significant difference between the BTV infection rate and the age of the animal. It was shown that the calves started to get infected with BTV after the age of 2 years. At this age, the animals are usually released onto the pasture for grazing, where they are likely to be exposed to infected vectors and subsequent BTV infection. We believe that the association of BTV infection rate and age is probably attributed to frequent exposure of older cattle to infected *Culicoides* vectors. In contrast, young calves (<2 years) are usually kept indoors and are well taken care of by the animal owners for protection against infectious diseases particularly, the insect and tick-borne infections [36]. Our result is in agreement with previous epidemiological surveys, which reported higher risks of older animals for BTV infections [37]. It should be noted that the BTV-specific antibodies detected among cattle in North Kordufan State indicate natural infection as there is no vaccination program for the disease in the country. In addition, all cattle included in this study are aged over one year. Therefore, it is assumed that detected antibodies no longer persisted and that antibody indicated direct exposure to BTV. In addition, there was association between body condition and BTV seropositivity. In our study, poor physical conditions are associated with highest prevalence of infection. It is probable that emaciated cattle have the tendency to attract the infected *Culicoides* insect vector of BTV. However, if the status of emaciation causes infection by BTV or vice versa needs further investigation and cannot be derived from this study. Regarding the management risk factors, there was a significant difference between the use of insecticide and the seropositivity to BTV infected cattle. It is, therefore, recommended that routine application of insecticide or insect repellents be considered to prevent BTV infection in susceptible livestock. In contrast, the risk assessment studies indicated that there was no significant difference between BTV infection and the rest of the individual or management risk factors included in the study. It is worth mentioning that gender has no significant difference for BTV infection among male and females as both sexes are equally infected with BTV. Likewise, there was no significant difference between localities and BTV infection rate, suggesting wide distribution of the *Culicoides* vector all over the localities of North Kordufan State. Highest and lowest rates of BTV positivity were recorded in the localities of Shiekan (15%) and Ennuhud (27.5%), respectively. The high level of BTV positivity in Ennuhud is attributed to the substantial rainfall events and development of irrigation projects, which provide suitable habitat for the insect vector in this locality. However, the locality of Shiekan is situated in the semi desert zone and hence has restricted rainfall.

## Conclusions

The result of this study indicated that BTV does exist in North Kordufan State, Sudan, and that susceptible livestock in the region are at risk of becoming infected with BTV. Nevertheless, an outbreak of BTV among native sheep in this region of the Sudan is yet to be reported. The BTV insect vectors and the specific virus serotypes circulating in the region remain to be identified. Future surveillance programs for BTV should be extended to include other susceptible animals such as sheep, goats and camels. In addition, the high-risk animal species (native sheep) and the distribution of *Culicoides* vectors in the region should also be considered to better predict and respond to a possible BTV outbreak in the State of North Kordufan, Sudan.

## Competing interests

The authors declare that they have no competing interests. The result of this study does not reflect the opinion of the funding sources.

## Authors’ contributions

IAA Collected blood samples, optimized the ELISA for detection of BTV-specific IgG antibodies in cattle sera, design of the experiment, and prepared the draft manuscript. MEHM and MAA designed the study. IEA designed the experiment and prepared the final manuscript. All authors read and approved the final version of the manuscript.

## Supplementary Material

Additional file 1 Investigation of Blue tongue virus infection among cattle in North Kordufan State, Sudan. Click here for file
