# Supplementary material for: Prevalence of bluetongue virus infection and associated risk factors among cattle in North Kordufan State, Western Sudan
Source: BMC Vet Res. 2014 Apr 24;10:94. doi: 10.1186/1746-6148-10-94 (PMC4022533; doi:10.1186/1746-6148-10-94)
Supplement: Additional file 1 — Investigation of Blue tongue virus infection among cattle in North Kordufan State, Sudan. [file 1746-6148-10-94-S1.doc]

**Questionnaire**

**Investigation of Blue tongue virus infection among cattle in North Kordufan State, Sudan**

**Date__________________ Locality _________________ Herd Owner___________________**

**Herd Code ____________________ Address ______________________________________**

**I. Individual risk factors**

**1-Age (years)**

Young <2 (   )

Old >2 (    )

**2-Sex**

Male (    )

Female (    )

**3-Breed**

Indigenous (    )

Cross (    )

**4-Body condition**

Emaciated  (    )

Fat (    )

**5- Source of animal**:

Raised on farm (    )

Purchased from other farms (    )

Purchased from local market (    )

**II. Management Risk Factors**

**6-Grazing system**

Nomadic (    )

Stationary (    )

**7-Herd size**

Small  (    )

Medium (    )

Large (    )

**8- Vector control**

Yes (    )

No (    )

**9.**- **Animal production**

Low ( )

High ( )

**10- Farm Yard**

Indoor ( )

Outdoor ( )

**11- Other animals in the herd**

**Yes ( )**

**No ( )**

**12- Locality**

1. AbuZabad (    ) 2. Umrawaba (    ) 3. Bara (    )

4. Sheikan (    ) 5. Ennuhud (    ) 6. Elkhuwaye ( )
